# The outcomes of Re-Redo bariatric surgery—results from multicenter Polish Revision Obesity Surgery Study (PROSS)

**DOI:** 10.1038/s41598-024-52817-7

**Published:** 2024-02-01

**Authors:** Michał Łabul, Michał Wysocki, Piotr Małczak, Maciej Matyja, Natalia Dowgiałło-Gornowicz, Paweł Lech, Michał Szymański, Piotr Major, PROSS–Collaborative Study Group, Michał Pędziwiatr, Justyna Rymarowicz, Piotr Zarzycki, Tomasz Stefura, Karol Ciszek, Piotr Myśliwiec, Hady Razak Hady, Paulina Głuszyńska, Monika Proczko-Stepaniak, Maciej Walędziak, Michał Janik, Andrzej Kwiatkowski, Magdalena Materlak, Katarzyna Bartosiak, Łukasz Czyżykowski, Maciej Mawlichanów, Piotr Kowalewski, Jacek Szeliga, Wojciech Kupczyk, Anna Harań, Grzegorz Kowalski, Rafał Mulek, Michał Kreft, Michał Orłowski, Paula Franczak, Artur Binda, Wiesław Tarnowski, Paweł Jaworski, Mateusz Kamiński, Maciej Pastuszka, Wojciech Lisik, Paweł Szymański, Bartosz Katkowski, Michał Leśniak

**Affiliations:** 1 Department of General Surgery, Specialist Hospital in Legnica, Legnica, Poland; 2 Department of General Surgery and Surgical Oncology, Ludwik Rydygier Memorial Hospital, Os. Złotej Jesieni 1, Cracow, Poland; 3 2nd Department of General Surgery, Jagiellonian University Medical College, Cracow, Poland; 4 Department of General, Minimally Invasive and Elderly Surgery, University of Warmia and Mazury in Olsztyn, Olsztyn, Poland; 5 Department of General, Endocrine and Transplant Surgery, Medical University of Gdansk, Gdańsk, Poland; 6 1st Department of General and Endocrinological Surgery, Medical University of Bialystok, Białystok, Poland; 7 Department of General, Oncological, Metabolic and Thoracic Surgery, Military Institute of Medicine, Warsaw, Poland; 8 Department of General, Gastroenterological, and Oncological Surgery, Collegium Medicum Nicolaus Copernicus University, Toruń, Poland; 9 Surgery Clinic Mazan, Katowice, Poland; 10 Department of General and Endoscopic Surgery, EuroMediCare Specialist Hospital and Clinic in Wroclaw, Wrocław, Poland; 11 Department of General and Oncological Surgery, Ceynowa Hospital, Wejherowo, Poland; 12 Department of General, Oncological and Digestive Tract Surgery, Centre of Postgraduate Medical Education, Orłowski Hospital, Warsaw, Poland; 13 Department of General, Transplant, Gastroenterological and Oncological Surgery, Medical University of Lodz, Lodz, Poland; 14 Department of General and Minimally Invasive Surgery, Łęczna, Poland; 15 Department of General and Transplantation Surgery, Medical University of Warsaw, Warsaw, Poland; 16 Department of General and Vascular Surgery, Polanica Zdrój, Poland

**Keywords:** Translational research, Endocrine system and metabolic diseases

## Abstract

The increasing prevalence of bariatric surgery has resulted in a rise in the number of redo procedures as well. While redo bariatric surgery has demonstrated its effectiveness, there is still a subset of patients who may not derive any benefits from it. This poses a significant challenge for bariatric surgeons, especially when there is a lack of clear guidelines. The primary objective of this study is to evaluate the outcomes of

patients who underwent Re-Redo bariatric surgery. We conducted a retrospective cohort study on a group of 799 patients who underwent redo bariatric surgery between 2010 and 2020. Among these patients, 20 individuals underwent a second elective redo bariatric surgery (Re-Redo) because of weight regain (15 patients) or insufficient weight loss, i.e. < 50% EWL (5 patients). Mean BMI before Re-Redo surgery was 38.8 ± 4.9 kg/m^2^. Mean age was 44.4 ± 11.5 years old. The mean %TWL before and after Re-Redo was 17.4 ± 12.4% and %EBMIL was 51.6 ± 35.9%. 13/20 patients (65%) achieved > 50% EWL. The mean final %TWL was 34.2 ± 11.1% and final %EBMIL was 72.1 ± 20.8%. The mean BMI after treatment was 31.9 ± 5.3 kg/m^2^. Complications occurred in 3 of 20 patients (15%), with no reported mortality or need for another surgical intervention. The mean follow-up after Re-Redo was 35.3 months. Although Re-Redo bariatric surgery is an effective treatment for obesity, it carries a significant risk of complications.

## Introduction

The demand for bariatric treatment has been steadily increasing over the years^1^. While the majority of patients who undergo bariatric surgery achieve satisfactory results and do not require additional interventions, the rise in the number of primary procedures has also led to a corresponding increase in the need for redo bariatric surgeries (RBS). This trend is expected to continue. Worldwide, RBS accounts for around 7% of all bariatric procedures, and in some countries like the U.S., it is the third most common type of bariatric surgery^2^. The most common reasons for RBS include weight regain, inadequate weight loss, and the need to control obesity-associated diseases^3^. In addition, less frequent factors such as gastroesophageal reflux disease (GERD), marginal ulcers, malnourishment, and fistulas can lead to RBS^4,5^. Like any invasive treatment, bariatric surgery is associated with certain complications including gastrointestinal bleeding, anastomotic leak, intestinal obstruction, dumping syndrome, nutritional deficiencies, GERD, biliary reflux and others^6,7^. According to meta-analysis by Chang et al. complication rates associated with bariatric surgery range from 10 to 17% and reoperation rates approximately at 7%; nonetheless, perioperative mortality is low (0.08–0.35%)^8^. RBS is generally reported to have higher complication rate compared to primary surgery^9–11^. Obesity is a chronic disease that can recur, and when a patient does not achieve the anticipated results after a secondary bariatric procedure, another surgery may be a potentially beneficial form of treatment. However, with a higher risk of complications and a scarcity of general guidelines, it is never an easy decision to qualify a patient for a third or subsequent bariatric operation.

## Purpose

The aim of this study is to evaluate patients who underwent Re-Redo bariatric surgery in terms of weight loss effectiveness and complications.

## Patients and methods

A retrospective cohort study was conducted in 12 referral bariatric centers in Poland analyzing consecutive patients who underwent redo surgical treatment for clinically severe obesity between January 2010 and January 2020. Participation in the study was voluntary. Entry criteria for bariatric centers to take part in the study was to report at least 30 RBS patients.

Inclusion criteria were: Re-Redo bariatric surgery after prior redo surgical treatment of obesity, laparoscopic approach, and patients ≥ 18 years. Bariatric operation following intragastric balloon treatment was not considered as a redo bariatric surgery. All patients were qualified and treated according to the commonly accepted guidelines^12,13^. The exclusion criteria were: RBS due to peri- or postoperative morbidity of primary procedure, lack of necessary data, and incomplete 12 months of bariatric follow-up after RBS. The primary endpoint was weight loss after Re-Redo and secondary endpoints were complications. Each participating bariatric center provided specific data, which were processed and used in the overall analysis.

**Figure 1:**
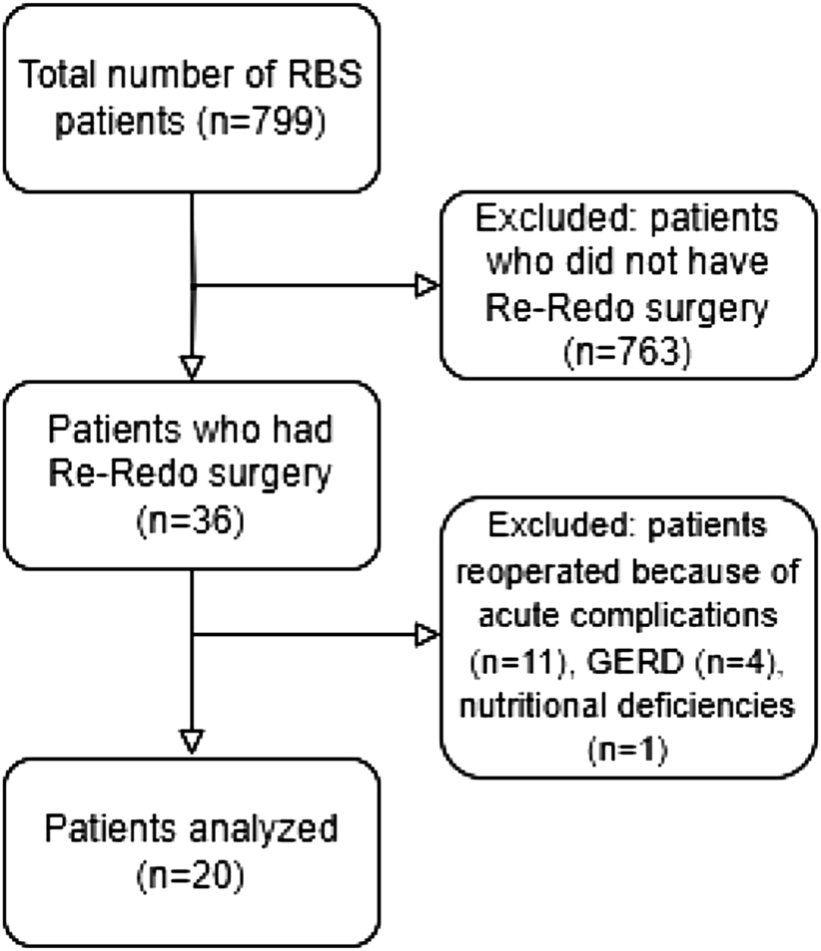
Flowchart of patients included in the study.

The study included 799 patients. 36 of them underwent Re-Redo bariatric surgery (Fig. 1). Of that group, 11 surgeries were emergency surgeries due to surgical complications such as fistulas (6), internal hernias (2), peritoneal abscess (1), leak from gastric remnant after one anastomosis gastric bypass (OAGB) (1), and persistent abdominal pain (1). Patients operated because of GERD (n = 4) and nutritional deficiencies (n = 1) were also excluded. In this paper we focus on the remaining 20 patients who underwent elective Re-Redo bariatric surgery because of weight regain or insufficient weight loss.

General characteristics of patients are presented in Table 1. Outcomes of PBS are presented in Table 2. Most of the study population were women (13 vs. 7). The mean age was 44.4 years old. The mean maximal body mass index (BMI) in the past was 49.4 kg/m^2^ and the mean BMI before primary bariatric procedure (PBS) was 47.5 kg/m^2^. Type 2 diabetes (T2D) was present in 7/20 patients (35%) and 13/20 patients (65%) had hypertension (HT). The median time interval between PBS and Redo treatments was 4 (2–6.5) years, and the median time interval between Redo and Re-Redo surgery was 3 (1–4.5) years. The mean follow-up after Re-Redo surgery was 35.3 months.

**Table 1:** General characteristics.

Males/females, n (%)	7/13 (35%/65%)
Age at the time of Re-Redo, years, mean ± SD (min–max)	44.4 ± 11.5 (19–68)
Maximal weight, kg, mean ± SD	143.7 ± 28.3
Maximal BMI, kg/m^2^, mean ± SD	49.4 ± 9.6
Tobacco smoking, n (%)	4 (20%)
Alcohol consumption, n (%)	7 (35%)
Weight before PBS, kg, mean ± SD	138.5 ± 25.0
BMI before PBS, kg/m^2^, mean ± SD	47.5 ± 7.9
Duration of obesity, n (%)	
< 5 years	1 (5%)
5–15 years	4 (20%)
> 15 years	15 (75%)
Type 2 diabetes, n (%)	7 (35%)
Hypertension, n (%)	13 (65%)
Asthma/COPD/OBS, n (%)	2 (10%)
Chronic heart failure, n (%)	1 (5%)
Preoperative intragastric balloon treatment, n (%)	1 (5%)

*BMI* body mass index, *PBS* primary bariatric surgery, *COPD* chronic obstructive pulmonary disease, *OBS* obstructive sleep apnea.

**Table 2:** PBS.

Median LOS, days, median (Q1–Q3)	3 (3–5)
Type of PBS, n (%)	
SG	3 (15%)
AGB	16 (80%)
Mason’s VBG	1 (5%)
Lowest weight after PBS, kg, mean ± SD	99.5 ± 25.8
Lowest BMI after PBS, kg/m^2^, mean ± SD	34.3 ± 8.8
%EBMIL, mean ± SD	62.0 ± 32.7
%TWL, mean ± SD	29.5 ± 16.3
Remission of T2D, n	1
Remission of hypertension, n	4

*PBS* primary bariatric surgery, *LOS* length of stay, *SG* sleeve gastrectomy, *AGB* adjustable gastric banding, *VBG* vertical banded gastroplasty, *%EBMIL* percentage of excess body mass index loss, *%TWL* percentage of total weight loss, *T2D* type 2 diabetes.

Compared with Redo group, Re-Redo patients were more likely to have hypertension. A full comparison of Redo and Re-Redo patients baseline conditions is included in the Supplement.

### Ethics statement

All procedures have been performed in accordance with the ethical standards laid down in the 1964 Declaration of Helsinki and its later amendments. Informed consent for surgical treatment was obtained from all patients before surgery. Protocol has been registered at clinical trials.gov (NCT05108532). There were no changes in treatment for patients included due to the study. The course of the study was closely monitored by a primary investigator who processed and verified any missing or unclear data submitted to the central database. The study was approved by the Bioethics Committee of the Regional Chamber of Physicians, District of Warmia and Mazury, Poland (23/2021/VIII).

## Results

Regarding their initial procedures, 16 patients had adjustable gastric banding (AGB), 3 had sleeve gastrectomy (SG), and one patient had Mason’s vertical banded gastroplasty. For their Redo surgery patients underwent SG (7 patients), Roux-en-Y gastric bypass (RYGB) (4), re-sleeve gastrectomy (3), replacement of adjustable gastric band (3), and gastric band removal (3).

Re-Redo bariatric surgery included SG (6), RYGB (5 patients), OAGB (3), and single anastomosis sleeve-ileal bypass (SASI) (2). The following procedures were performed only once: biliopancreatic diversion with duodenal switch (BPD-DS), AGB after RYGB, biliopancreatic limb lengthening after RYGB, gastrojejunal reanastomosis with biliopancreatic limb lengthening after RYGB.

Among the reasons for Redo surgery were insufficient weight loss i.e. < 50% excess weight loss (EWL) (6 patients), weight regain (4), and band slippage and/or erosion (10). Of these patients, 15 underwent Re-Redo surgery because of weight regain and 5 due to insufficient weight loss < 50% EWL.

Results of Re-Redo bariatric surgery are presented in Table 3.

**Table 3:** Re-Redo surgery.

Reasons for Re-Redo surgery, n	
< 50%EWL	5 (25%)
Weight regain	15 (75%)
Treatment in bariatric center that performed Redo surgery, n (%)	16 (80%)
Weight before Re-Redo, kg, mean ± SD	113.0 ± 16.3
BMI before Re-Redo, kg/m^2^, mean ± SD	38.8 ± 4.9
%EBMIL before Re-Redo, mean ± SD	38.3 ± 23.1
%TWL before Re-Redo, mean ± SD	19.5 ± 13.8
Time interval between PBS and Re-Redo, years, median (q1–q3)	7.5 (4–10)
Time interval between Redo and Re-Redo, years, median (q1–q3)	3 (1–4.5)
LOS, days, median (q1–q3)	3 (3–4)
Type of Re-Redo, n (%)	
SG	6 (30%)
RYGB	5 (25%)
OAGB	3 (15%)
SASI	2 (10%)
Biliopancreatic limb lengthening (after RYGB)	1 (5%)
Gastrojejunal reanastomosis with biliopancreatic limb lenghtening (after RYGB)	1 (5%)
AGB (after RYGB)	1 (5%)
BPD-DS	1 (5%)
Final weight after Re-Redo, kg, mean ± SD	92.7 ± 17.8
Final BMI after Re-Redo, kg/m^2^, mean ± SD	31.9 ± 5.3
Final %EBMIL, mean ± SD	72.1 ± 20.8
Final %TWL, mean ± SD	34.2 ± 11.1
%EBMIL regarding BMI before and after Re-Redo, mean ± SD	51.6 ± 35.9
%TWL regarding BMI before and after Re-Redo, mean ± SD	17.4 ± 12.4
Complications after Re-Redo, n (%)	3 (15%)
Vomiting more often than once a week	1 (5%)
Esophagitis, nutricional deficiencies	1 (5%)
Persistent abdominal pain complaints, episode of biliary colic, GERD	1 (5%)
Remission of T2D, n	4
Remission of hypertension, n	0

*GERD* gastroesophageal reflux disease, *%EWL* percentage of excess weight loss, *BMI* body mass index, *%EBMIL* percentage of excess body mass index loss, *%TWL* percentage of total weight loss, *PBS* primary bariatric surgery, *LOS* length of stay, *OAGB* one anastomosis gastric bypass, *RYGB* Roux-en-Y gastric bypass, *SASI* single anastomosis sleeve-ileal bypass, *SG* sleeve gastrectomy, *AGB* adjustable gastric banding, *BPD-DS* biliopancreatic diversion with duodenal switch, *T2D* type 2 diabetes.

### Weight loss

**Figure 2:**
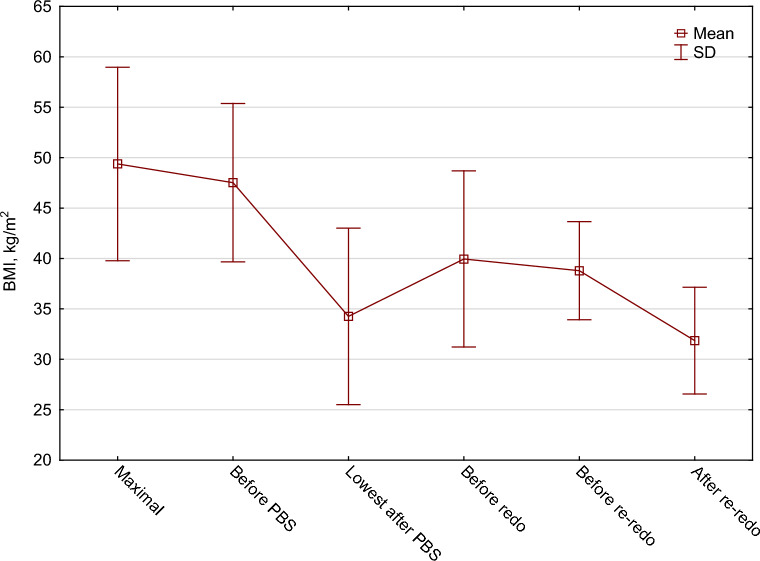
Changes in patients’ BMI.

**Figure 3:**
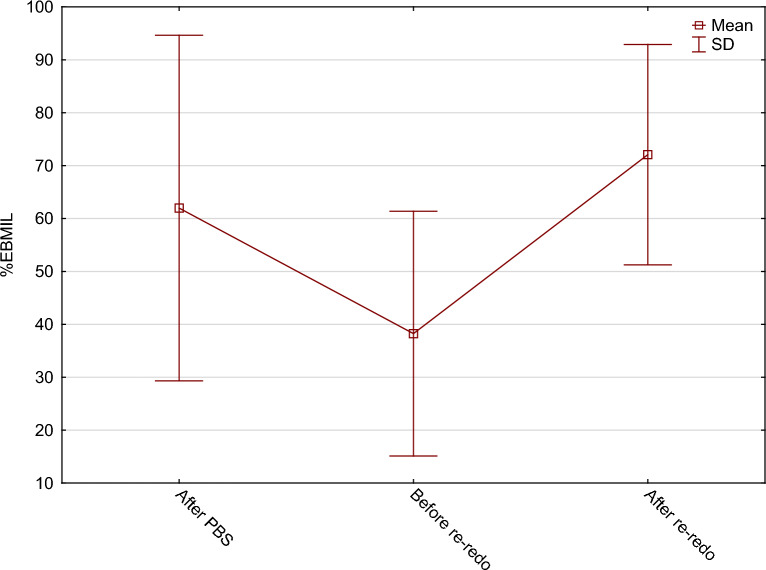
Changes in patients’ %EBMIL.

**Figure 4:**
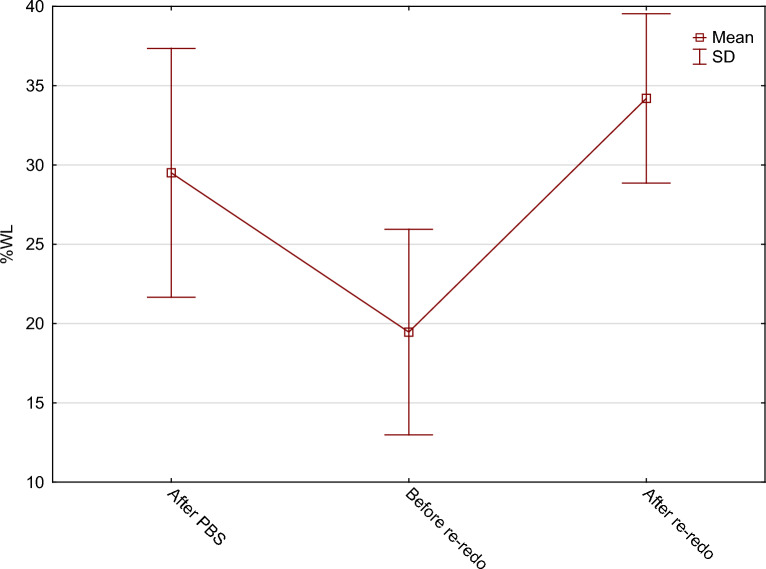
Changes in patients’ %TWL.

The mean percentages of excess body mass index loss (%EBMIL) were consecutively: 62.0 ± 32.7% after PBS, 38.3 ± 23.1% before Re-Redo, and 72.1 ± 20.8% after Re-Redo surgery. The mean percentage of total weight loss (%TWL) were: 29.5 ± 16.3% after PBS, 19.5 ± 13.8% before Re-Redo and 34.2 ± 11.1% after Re-Redo surgery. %TWL and %EBMIL regarding BMI before and after Re-Redo was 17.4 ± 12.4% and 51.6 ± 35.9% consecutively. Changes in patients’ BMI throughout the entire treatment are presented in Fig. 2. Changes in %EBMIL and %TWL are shown in Figs. 3 and 4.

The mean lowest BMI after PBS was 34.3 kg/m^2^ and the mean highest %TWL after PBS was 29.5%.

The mean BMI before Redo surgery was 40.0 ± 8.7 kg/m^2^. The mean BMI before Re-Redo surgery was 38.8 ± 4.9 kg/m^2^. The mean final BMI after Re-Redo was 31.9 ± 5.3 kg/m^2^.

### Complications

Complications after Re-Redo surgery occurred in 3 of 20 patients (15%). None of them required further surgical intervention. Among these complications were:

1. Vomiting more than once a week—a patient who had SG as PBS, reSG as Redo because of weight regain and OAGB as Re-Redo because of insufficient weight loss (< 50%EWL).
2. Esophagitis and nutritional deficiencies—a patient who had AGB as PBS, band removal with simultaneous SG as Redo because of gastric band intolerance (nausea, pain after eating) and RYGB as Re-Redo because of weight regain and also GERD.
3. Persistent abdominal pain complaints, episode of biliary colic, GERD—a patient who had AGB as PBS, gastric band removal as Redo because of band dysfunction along with GERD-associated symptoms and SG as Re-Redo because of weight regain.

From our group, 5 of 20 patients experienced complications after the first Redo surgery. Details are provided in Table 4. This is a similar complication rate compared to all the patients who underwent Redo surgery included in PROSS, which was 27.8% (222/799); the most common complications included GERD—117 (14.64%) patients, followed by vomiting in 42 (5.26%) cases, band malfunction in 20 (2.5%) patients, gastrointestinal obstruction in 20 (2.5%) patients, malnutrition in 9 (1.12%) patients, gastrointestinal leakage in 5 (0.63%) patients, and anemia in 4 (0.5%) patients^14^.

**Table 4:** Redo surgery.

Reasons for Redo surgery, n (%)	
< 50%EWL	6 (30%)
Weight regain	4 (20%)
Band slippage/erosion	10 (50%)
Treatment in bariatric center that performed PBS, n (%)	14 (70%)
Weight before Redo, kg, mean ± SD	116.0 ± 24.9
BMI before Redo, kg/m^2^, mean ± SD	40.0 ± 8.7
Time interval between PBS and Redo, years, median (q1–q3)	4 (2–6.5)
Type of Redo, n (%)	
ReSG	3 (15%)
ReAGB	3 (15%)
SG	7 (35%)
RYGB	4 (20%)
Gastric band removal	3 (15%)
Complications of Redo surgery, n (%)	5 (25%)
GERD	3 (15%)
Gastric band slippage	2 (10%)
Remission of T2D, n	1
Remission of hypertension, n	4

*%EWL* percentage of excess weight loss, *GERD* gastroesophageal reflux disease, *PBS* primary bariatric surgery, *BMI* body mass index, *OAGB* one anastomosis gastric bypass, *ReSG* re-sleeve gastrectomy, *ReAGB* replacement of adjustable gastric band, *SG* sleeve gastrectomy, *RYGB* Roux-en-Y gastric bypass, *T2D* type 2 diabetes.

## Discussion

Some patients undergo multiple bariatric operations in their lifetime. In this study we showed that Re-Redo bariatric surgery yields good results in terms of weight loss, but it might be associated with a higher risk of complications.

Bariatric surgery is a well-established obesity treating method yielding excellent outcomes, but approximately 15–20% of patients ultimately do not achieve or sustain satisfactory weight loss^15^. In addition, some patients may experience side effects of their bariatric surgery, such as GERD, which in some cases require surgical correction^16–18^.

RBS is a treatment option for those who do not achieve sufficient weight loss after their primary bariatric procedure, but it is more challenging and associated with a higher risk of complications than primary bariatric surgery PBS. Consequently, it seems appropriate for RBS to be performed at centers experienced in these types of procedures^19–21^.

Evidence-based publications, such as meta-analysis and systematic reviews by Brethauer et al.^22^, Koh et al.^3^, and Kermansaravi et al.^23^, do support the efficacy of RBS. A trial by Sudan et al. on over 28,000 patients demonstrated RBS to be safe, with 1.9% of severe adverse events and 0.24% mortality at 1 year for corrective operations, and respectively 3.61% and 0.31% in regards to conversions procedures^24^. Studies by multicenter Polish Revision Obesity Surgery Study (PROSS) also support these arguments^14,25^.

The literature on efficacy and safety of Re-Redo bariatric surgery is sparse and based on small groups of patients. We found four studies on this subject, with the number of analyzed patients ranging from 12 to 42, mean %EWL from 43.3 to 54.4% and complication rate from 14.7 to 35.7%^26–29^.

Paper by Daigle et al. on 12 patients found mean BMI after Re-Redo surgery to be 39.9 ± 20.8 kg/m^2^ and mean %EWL 54.4 ± 44.0%. 5 early complications occurred in 4 patients (33.3%), from which 2 needed operative intervention: partial gastrectomy of a necrotic gastric remnant after RYGB and mesh explantation after Roux limb lengthening with complex hernia repair. Other complications were wound infections^26^.

In study by Lunel et al. on 34 patients, final BMI was 36.8 ± 8.0 kg/m^2^ and %EWL after Re-Redo surgery was 47.9 ± 32.1%. Three patients (8.8%) presented severe complication, from which two of them was diagnosed with a leak of duodenoileal anastomosis after BPD-DS, and one had heavy malnutrition after reSG and required a jejunostomy. Another two patients (5.9%) experienced minor complications, one each of pneumonia and wound abscess^27^.

Study by Raglione et al. on 30 patients showed %EWL of 53.4% and %TWL of 29.6%. The complication rate was 30%. 3 patients were recognized with early postoperative leakage; treated endoscopically or with CT scan-guided drainage. 2 patients had postoperative bleeding that needed blood transfusion. 2 patients experienced dumping syndrome managed with dietary changes and acarbose. 2 patients developed late gastrojejunostomy stricture after RYGB treated successfully with endoscopic dilatations. None of these complications required another surgical intervention^28^.

Work by Nevo et al. evaluated 42 patients that underwent RYGB as a third or subsequent bariatric surgery. From this group, 32 patients had RYGB as their Re-Redo surgery. Mean final BMI reached 34.5 kg/m^2^ reflecting an excess BMI loss (%EBMIL) of 43.3%. Complication rate was 35.7%, but majority of it (10/15) were minor (Clavien-Dindo II). 5 patients needed reoperation, although 2 of these were negative explorations in suspected leak. Other reasons for reoperations included jejunojejunostomy stricture, small bowel obstruction due to adhesions, and anastomotic intraluminal bleeding^29^.

In our study, based on weight measured before and after Re-Redo, the mean %TWL was 17.4% and %EBMIL was 51.6%. 13/20 patients (65%) achieved > 50% EWL. Final %TWL was 34.2 ± 11.1%. Final BMI was 31.9 ± 5.3 kg/m^2^. Mean final %EBMIL at the end of treatment was 72.1 ± 20.9%. Complications occurred in 3 of 20 patients (15%), one each of the following: vomiting more than once a week, esophagitis and nutritional deficiencies, abdominal pain complaints with episode of bilary colic, and GERD. Each of these complications were treated successfully with dietary intervention and pharmacotherapy. None of them required additional surgical intervention. There was no mortality within 30 days of Re-Redo. In terms of weight loss effectiveness and the frequency of complications, the results of our study agree with the results of the studies mentioned above.

This study has several limitations. Our results are not confronted with any other different therapeutic options, in particular those regarding non-surgical treatment. This is a non-randomized study, however, it can be extremely difficult to perform a randomized trial here. The study covered a small group of patients, but this is mainly due to the fact that such patients are rare, although their number will most likely increase over time. There is no standardization of the surgeries performed on the patients included in the study. Patients were operated by different surgeons from 12 bariatric centers. However, RBS are often performed in different surgical centers by different surgical teams and tailored individually to a patient. For this reason and due to the lack of generally accepted guidelines for RBS, full standardization may be difficult to achieve.

## Conclusion

Re-Redo bariatric surgery is an effective treatment that should be considered in patients with weight regain or unsatisfactory weight loss after previous bariatric surgeries, but it comes with a considerable risk of complications. It's important to evaluate each patient on a case-by-case basis and not deny them assistance solely based on their previous bariatric treatment. Referral centers should be the optimal choice for performing RBS due to its higher complexity and the potential for complications.

Despite its potential benefits, there's still limited clinical evidence on Re-Redo bariatric surgery. Initial data appears promising, but more research is needed to gain a better understanding of its effectiveness and possible risks.

### Supplementary Information

Supplementary Information.

## Data Availability

The datasets used and/or analysed during the current study available from the corresponding author on reasonable request.
