# Supplementary material for: The outcomes of Re-Redo bariatric surgery—results from multicenter Polish Revision Obesity Surgery Study (PROSS)
Source: Sci Rep. 2024 Feb 1;14:2699. doi: 10.1038/s41598-024-52817-7 (PMC10834407; doi:10.1038/s41598-024-52817-7)
Supplement: Supplementary file 1 — Supplementary Information. [file 41598_2024_52817_MOESM1_ESM.docx]

| Table 1. Results of post-hoc Bonferroni’s test after repeated measurements ANOVA for BMI. P-value of repeated measurements ANOVA = 0.001. | | | | | | |
| --- | --- | --- | --- | --- | --- | --- |
|  | Highest BMI before treatment | BMI before treatment | Lowest BMI after PBS | BMI before Redo | BMI before Re-Redo | BMI at the end of observation |
| Highest BMI before treatment |  | 0.999 | <0.001 | <0.001 | <0.001 | <0.001 |
| BMI before treatment | 0.999 |  | <0.001 | <0.001 | <0.001 | <0.001 |
| Lowest BMI after PBS | <0.001 | <0.001 |  | 0.017 | 0.139 | 0.999 |
| BMI before Redo | <0.001 | <0.001 | 0.017319 |  | 0.999 | <0.001 |
| BMI before Re-Redo | <0.001 | <0.001 | 0.139 | 0.999 |  | 0.001 |
| BMI at the end of observation | <0.001 | <0.001 | 0.999 | <0.001 | 0.001 |  |

| Table 2. Results of post-hoc Bonferroni’s test after repeated measurements ANOVA for %EBMIL. P-value of repeated measurements ANOVA = 0.001. | | | |
| --- | --- | --- | --- |
|  | EBMIL after PBS | EBMIL between highest BMI and BMI before Re-Redo | EBMIL between highest BMI and BMI at the end of observation |
| EBMIL after PBS |  | 0.012 | 0.613 |
| EBMIL between highest BMI and BMI before Re-Redo | 0.012 |  | <0.001 |
| EBMIL between highest BMI and BMI at the end of observation | 0.613 | <0.001 |  |

| Table 3. Results of post-hoc Bonferroni’s test after repeated measurements ANOVA for %TWL. P-value of repeated measurements ANOVA = 0.001. | | | |
| --- | --- | --- | --- |
|  | TWL after PBS | TWL between highest BMI and BMI before Re-Redo | TWL between highest BMI and BMI at the end of observation |
| TWL after PBS |  | 0.014 | 0.518 |
| TWL between highest BMI and BMI before Re-Redo | 0.014 |  | <0.001 |
| TWL between highest BMI and BMI at the end of observation | 0.518 | <0.001 |  |

| Table 4. Results of univariate logistic regression models analyzing potential risk factors for re-redo based on basic characteristics of general population of PROSS study group | | | | | |
| --- | --- | --- | --- | --- | --- |
|  | | OR | -95%CI | +95%CI | p-value |
| Females | | 0.614 | 0.278 | 1.356 | 0.227 |
| Age | | 1.006 | 0.969 | 1.045 | 0.754 |
| Maximal lifetime BMI | | 1.012 | 0.970 | 1.057 | 0.576 |
| Smoking | | 0.904 | 0.259 | 3.155 | 0.874 |
| Alcohol consumption | | 1.193 | 0.477 | 2.985 | 0.706 |
| BMI before PBS | | 1.013 | 0.972 | 1.056 | 0.539 |
| Duration of obesity | < 5 years | 1.00 | Ref. | Ref. |  |
|  | 5-15 years | 1.241 | 0.155 | 9.947 | 0.849 |
|  | > 15 years | 1.739 | 0.223 | 13.579 | 0.598 |
| Type 2 diabetes mellitus | | 1.471 | 0.666 | 3.248 | 0.339 |
| Hypertension | | 2.424 | 1.080 | 5.437 | **0.032** |
| Asthma/OSA/COPD | | 1.015 | 0.234 | 4.411 | 0.984 |

List of bariatric centers that participated in the study:

1. 2nd Department of General Surgery, Jagiellonian University Medical College, Krakow, Poland

2. Department of General Surgery and Surgical Oncology, Ludwik Rydygier Memorial Hospital, Krakow, Poland

3. Department of General and Endoscopic Surgery, EuroMediCare Specialist Hospital and Clinic, Wroclaw, Poland

4. Department of General and Endocrine Surgery, Medical University of Bialystok, Bialystok, Poland

5. Department of General, Endocrine and Transplant Surgery, Medical University of Gdansk, Gdansk, Poland

6. Surgery Clinic Mazan, Katowice, Poland

7. Department of General and Oncological Surgery, Ceynowa Hospital, Wejherowo, Poland

8. Department of General, Gastroenterological, and Oncological Surgery, Collegium Medicum Nicolaus Copernicus University, Torun, Poland

9. Department of General, Oncological and Digestive Tract Surgery, Centre of Postgraduate Medical Education, Orłowski Hospital, Warsaw, Poland

10. Department of General, Minimally Invasive and Elderly Surgery, University of Warmia and Mazury, Olsztyn, Poland

11. Department of General, Oncological, Metabolic and Thoracic Surgery, Military Institute of Medicine, Warsaw, Poland

12. Department of General and Vascular Surgery, Polanica Zdroj, Poland
